# Amphiregulin promotes hair regeneration of skin‐derived precursors via the PI3K and MAPK pathways

**DOI:** 10.1111/cpr.13106

**Published:** 2021-08-12

**Authors:** Qiumei Lu, Ying Gao, Zhimeng Fan, Xing Xiao, Yu Chen, Yuan Si, Deqiang Kong, Shuai Wang, Meijian Liao, Xiaodong Chen, Xusheng Wang, Weiwei Chu

**Affiliations:** ^1^ School of Pharmaceutical Sciences (Shenzhen) Sun Yat‐sen University Guangzhou China; ^2^ Department of Anesthesiology The First People’s Hospital of Foshan Foshan China; ^3^ School of Life Sciences Tsinghua University Beijing China; ^4^ Center of Scientific Research The Seventh Affiliated Hospital of Sun Yat‐sen University Shenzhen China; ^5^ Department of Dermatology Guangzhou First People's Hospital Guangzhou China; ^6^ The Yonghe Medical Beauty Clinic Limited Company Guangzhou China; ^7^ School of basic medicine Xuzhou Medical University Xuzhou China; ^8^ Department of Burn Surgery The First People’s Hospital of Foshan Foshan China

**Keywords:** 3D co‐culture system, amphiregulin, hair follicle, MAPK, PI3K, skin‐derived precursors

## Abstract

**Objectives:**

There are significant clinical challenges associated with alopecia treatment, including poor efficiency of related drugs and insufficient hair follicles (HFs) for transplantation. Skin‐derived precursors (SKPs) exhibit great potential as stem cell‐based therapies for hair regeneration; however, the proliferation and hair‐inducing capacity of SKPs gradually decrease during culturing.

**Materials and Methods:**

We describe a 3D co‐culture system accompanied by kyoto encyclopaedia of genes and genomes and gene ontology enrichment analyses to determine the key factors and pathways that enhance SKP stemness and verified using alkaline phosphatase assays, Ki‐67 staining, HF reconstitution, Western blot and immunofluorescence staining. The upregulated genes were confirmed utilizing corresponding recombinant protein or small‐interfering RNA silencing in vitro, as well as the evaluation of telogen‐to‐anagen transition and HF reconstitution in vivo.

**Results:**

The 3D co‐culture system revealed that epidermal stem cells and adipose‐derived stem cells enhanced SKP proliferation and HF regeneration capacity by amphiregulin (AREG), with the promoted stemness allowing SKPs to gain an earlier telogen‐to‐anagen transition and high‐efficiency HF reconstitution. By contrast, inhibitors of the phosphoinositide 3‐kinase (PI3K) and mitogen‐activated protein kinase (MAPK) pathways downstream of AREG signalling resulted in diametrically opposite activities.

**Conclusions:**

By exploiting a 3D co‐culture model, we determined that AREG promoted SKP stemness by enhancing both proliferation and hair‐inducing capacity through the PI3K and MAPK pathways. These findings suggest AREG therapy as a potentially promising approach for treating alopecia.

## Graphical abstract

The 3D co‐culture system reveals that Epi‐SCs and ASCs help SKPs express a higher level of AREG, leading to upregulation of the downstream PI3K and MAPK pathways. As a result, SKP capabilities of proliferation and HF regeneration are promoted.

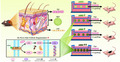

## 1 INTRODUCTION

Alopecia resulting from various factors that decrease hair follicle (HF) regeneration^1^ affects a significant fraction of the world population. Although not life‐threatening, hair loss predisposes people to considerable psychological distress^2^, ^3^ and severe quality‐of‐life impairment.^4^ Its pathogenesis varies and leads to symptoms, such as androgenetic alopecia, alopecia areata, diffuse alopecia and therapy‐induced hair loss.^5^ Several oral, surgical, topical and intralesional treatments have been progressively developed in recent decades to achieve delayed hair loss or hair restoration in alopecic areas.^6^

Although hair transplantation is regarded as the gold standard for creating natural‐appearing hair in androgenic alopecia (AGA),^7^ there is an unavoidable controversy surrounding autologous transplantation owing to the limited source of donor hair, the decreased viability of cells extracted in this time‐consuming procedure, and, most importantly, the impermanent outcomes as the disease progresses.^1^, ^3^ Regarding oral medicines, drugs approved by the Food and Drug Administration (FDA), such as finasteride, dutasteride and minoxidil for AGA, vary greatly in efficacy from person to person and provide partial and temporary benefits, as well as side effects.^8^, ^9^, ^10^, ^11^ Such surgical procedures and medications cannot always meet patient satisfaction given the finite sources, unfavourable side effects and partial benefits.

Stem cell‐based therapies have gained tremendous attention owing to their advantages of infinite self‐renewal capacity and multi‐lineage differential potential,^6^ thereby providing approaches to coping with the challenges posed by traditional alopecia therapies. Mesenchymal stem cells (MSCs) show considerable promise for intravenous injection of human MSCs into non‐scarring alopecia^12^ and co‐culture with human dermal papilla cells (hDPCs)^13^, ^14^ in preclinical studies. Moreover, using conditioned medium from cells (eg, Wnt1a,^15^, ^16^ Wnt7a,^17^ or Nanog‐overexpressing MSCs^18^) can accelerate the telogen‐to‐anagen transition of HFs.^19^ Adipose‐derived stem cells (ASCs) are another latent cell population used in regenerative medicine. With their advantage of being easier to obtain than MSCs and low immunogenicity, they play a vital role in the activation of epidermal stem cells or hDPCs by secreting growth factors, such as vascular endothelial growth factor, hepatocyte growth factor, platelet‐derived growth factor, insulin‐like growth factor 1, thymosin beta 4, stromal cell‐derived factor 1α, proinflammatory chemokine C–C motif chemokine ligand 2 and endothelial growth factor, thereby providing significant promotion of HF morphogenesis both in vitro and in vivo.^20^, ^21^, ^22^, ^23^, ^24^, ^25^, ^26^, ^27^, ^28^ Additionally, administering the stromal vesicular fraction (SVF) or lipoaspirate obtained from abdominal fat to the scalp graft yields favourable outcomes.^29^, ^30^ However, MSC and ASC therapies utilize the paracrine function, and the implanted cells cannot regenerate into HFs. Accordingly, considering that the treatment effect is not guaranteed for different types and severity of hair loss and their inability to naturally grow and differentiate into HFs specifically, identification alternate and more promising strategies for hair regeneration is required.

Skin‐derived precursors (SKPs) are a multipotent precursor cell population from the adult mammalian dermis capable of differentiating into several lineages, such as dermal, neural and mesodermal progeny and show promise for therapeutic and regenerative medicine associated with HFs.^31^, ^32^, ^33^, ^34^ The DP of HFs appears to comprise SKPs based on identical patterns of gene expression (Nexin, Wnt5a and versican), and cells from adult whisker follicle papillae cultivated under SKP conditions can generate properties similar to SKPs.^35^ SKPs from the HF niche can not only differentiate into dermal cell types, such as fibroblasts and myofibroblasts, but also induce HF morphogenesis.^36^, ^37^ These findings indicate SKPs as ideal “seed cells” to yield complete HF structures,^38^ thereby providing a possible avenue to address the graft origin of hair loss.

However, following SKP extraction from their physiological environment, their ability to generate large amounts or functional HFs decreases over time. Furthermore, the critical signalling pathways necessary to maintain SKP properties, self‐renewal and proliferation remain unclear. Methods to produce highly proliferative SKPs capable of modulating cell fate in HF generation are required. Therefore, we used a 3D co‐culture model, where distinct cell types were cultured within the same confined environment to simulate the microenvironment in vivo, in order to maintain their proliferation and HF‐formation potency.^39^, ^40^, ^41^ In the present study, SKPs were co‐cultured with ASCs and epidermal stem cells (Epi‐SCs) so as to imitate the surrounding niche of SKPs in the dermal layer of the skin and identify the crucial factors and pathways in the co‐culture system. Furthermore, this model allowed the exploration of critical targets for preserving dermal stem cell‐proliferative capacity.

## 2 MATERIALS AND METHODS

### 2.1 Isolation and culture of SKPs and epidermal keratinocytes from skin

All experiments involving live rodents conformed to appropriate governmental and institutional regulations and were performed according to the guidelines of the Animal Ethics Committee of Sun Yat‐sen University (approval no. SYSU‐YXYSZ‐20210332). C57BL/6 and BALB/c ^nu/nu^ mice were obtained from the Laboratory Animal Center of Sun Yat‐sen University (Guangzhou, China). Dorsal skin from CO_2_‐asphyxiated postnatal day 2 (P2) juvenile C57BL/6 mice were dissected, washed with phosphate‐buffered saline (PBS; Biofil Chemicals and Pharmaceuticals Ltd.) and cut into pieces (2‐3 mm^2^) that were digested with 0.35% dispase II (Sigma‐Aldrich) at 4°C overnight on a rotator. After washing twice with PBS, one pair of forceps was used to hold down a corner of the dermis, and the other was used to gently lift the epidermis away from the dermal sheet. Sheared as finely as possible, Epi‐SCs were released into a 0.035% collagenase I (Sigma‐Aldrich) solution, and dermal cells from the dermal layer were digested in a 0.35% collagenase I solution at 37°C for 2 hours. After incubation, digestion enzyme activity was neutralized with an equal volume of Dulbecco's modified eagle medium (DMEM) containing 20% foetal bovine serum (FBS). The epidermal and dermal cell suspensions were then separately passed through two 100‐μm cell strainers (Biofil) and centrifuged for 8 minutes at 1400 rpm at room temperature (RT). After aspirating the supernatant, dermal cells were resuspended in red blood cell lysis buffer (Sigma‐Aldrich) for 5 minutes and centrifuged for 5 minutes at 1400 rpm at RT. Epi‐SCs were resuspended in defined keratinocyte serum‐free medium (Gibco), changed every other week and transferred into the inserts of a 12‐well transwell plate. Additionally, the dermal cells were resuspended in DMEM‐F12 (3:1; Invitrogen) containing 20 ng/mL epidermal growth factor (EGF; Peprotech), 40 ng/mL fibroblast growth factor (FGF; Peprotech) and 2% B27 (Gibco) at a density of 1 × 10^5^ cells/mL in a tissue culture (TC)‐treated petri dish for 8 hours until some cells adhered to the bottom (for ease of description, we refer to the medium for the dermal cells as DMEM‐EFB). Purified populations of SKPs were obtained via selective adhesion. To be specific, adherent cells were discarded, and non‐adherent cells were cultured. After transfer to 0.001% (w/v) Pluronic F‐127 (Sigma‐Aldrich; PF127) coated 6‐well plates, the small floating spheres proliferated to larger spheres in the same medium as the initial culture (DMEM‐EFB). EGF, FGF and B27 were supplemented at the initial concentrations every 3 days. When passaged every 6 days, the spheres (dissociated to single cells by pre‐warmed TrypLE Express [Gibco]) would subsequently generate new spheres under the same culture conditions.^42^

### 2.2 Isolation of ASCs

The CO_2_‐asphyxiated 9‐week‐old C57BL/6 mice were positioned in dorsal recumbency. Areas to be incised for harvesting adipose tissue need to be depilated entirely to avoid hair contamination. Adipose tissue from the subcutaneous and inguinal tissues was meticulously dissected and harvested. The linea alba was then longitudinally incised down to the peritoneal cavity in order to expose numerous locations for harvesting adipose tissue. The harvesting procedure was completed within 20 minutes of animal sacrifice.

The harvested adipose tissue was placed in a TC‐treated petri dish and finely minced into small pieces with a pair of sterile surgical scissors. The tissue was then rinsed with PBS containing 2% penicillin/streptomycin (Gibco) solution, and the sample was covered with 0.1% collagenase I solution and incubated at 37°C at 250 rpm for 1 hour. DMEM containing 10% FBS was then added to inactivate collagenase I, followed by centrifugation at 2000 rpm for 5 minutes to obtain a high‐density pellet (constituting the SVF, separated from the remaining fat lobules and oil). The pelleted SVF was then resuspended in red blood cell lysis buffer for 5 minutes at RT to lyse contaminating red blood cells, collected by centrifugation and filtered through a 100‐μm nylon mesh to remove cell debris. After washing with PBS, the SVF was again passed through a 70‐μm cell strainer (Biofil) and centrifuged at 2000 rpm for 5 minutes. ASCs were then cultured in low‐glucose DMEM (Gibco) containing 15% FBS, 1% L‐glutamine (Gibco) and 1% penicillin/streptomycin solution. When the primary cells reached 80% to 90% confluence following changes in medium every 2 days, they were trypsinized and passaged in typical 6‐well plates at a 1:3 dilution. P2 to P5 cells were used for in vivo and in vitro assays.^43^, ^44^

### 2.3 3D co‐culture system

P3 SKPs were cultured alone at a density of 5 × 10^4^ cells/well in the 0.001%PF127‐precoated middle compartment of the 6‐well transwell co‐culture dishes in the control group. For the dual‐cell 3D co‐culture system, SKPs (P3) were added to the 0.001%PF127‐precoated middle compartment of the culture dish, whereas ASCs (5 × 10^4^ cells/well) or Epi‐SCs (2.5 × 10^4^ cells/well) were seeded in the lower or upper compartment, as shown in the graphical abstract. Regarding the ternary‐cell 3D co‐culture system, Epi‐SCs, SKPs (P3) and ASCs were separately cultured in the upper, middle and lower compartments of the transwell culture dish, respectively. Two inserts separated the well into three compartments with permeable membranes (the upper insert: 1‐μm pore size, 12‐well format, polyethylene terephthalate [PET], cat no. 353103; and the lower insert: 1‐μm pore size, 6‐well format, PET, cat no. 353102; Corning Inc) on the bottom and uniquely stacked into each other. This plate was inserted in the incubator on a shaker, and the 1‐μm‐diameter pores in the porous membrane allowed molecules to diffuse between the cell layers without permitting cell‐cell contact. The cells were co‐cultured for 6 days, with the medium changed on day 3.

### 2.4 RNA interference

Small‐interfering (si) RNA was used for amphiregulin (AREG) knockdown. Duplexes of three oligos targeting *AREG* and the scrambled carboxyfluorescein (FAM)‐labelled oligo serving as the negative control (NC) (Table S1) were designed using the siRNA selection programme from the Whitehead Institute (http://sirna.wi.mit.edu/) and synthesized by GenePharma. The most efficacious siRNA among the three targeting siRNAs was used in subsequent experiments. SKPs (P3) were collected from P2 C57BL/6 mice and seeded at a density of 2.5 × 10^6^ cells/mL (2 mL/well) in 6‐well culture plates 6 h before transfection. GP‐transfect‐Mate (16 μL, diluted in 200 μL Opti‐MEM) was used to transfect siRNA (200 nmol/L, diluted in 200 μL Opti‐MEM) into SKPs for 48 hours according to manufacturer instructions. The NC‐FAM group was observed by fluorescence microscopy to verify transfection efficiencies of >70% at 24‐h post‐transfection. The medium was changed to remove transfection reagents at 48‐h post‐transfection, and SKPs were subjected to Western blot at 72‐h post‐transduction.

### 2.5 Alkaline phosphatase (AP) colour‐development experiment

Skin‐derived precursors were allowed to attach the bottom by replacing the medium with DMEM containing 10% FBS and the PF127‐treated plate with a regular TC‐treated plate. After 6 hours of incubation, the attached SKPs were rinsed with PBS, fixed with 4% paraformaldehyde (PFA; Solarbio) at RT for 10 minutes and washed again with PBS. The cells were then incubated for 1 hour with BCIP/NBT solution (prepared from Fast BCIP/NBT buffered substrate tablet; Sigma‐Aldrich), and the colour development was stopped by rinsing with distilled water. Finally, the depth of the purple colonies was observed under a light microscope (Nikon). Similar to other lineages and development‐specific genes recognized as a determinant to stem cells, AP levels during ontogenesis in some tissue regions also correspond to those of particular stem cell precursors and their niches. Thus, high AP activity is a unique and unambiguous biomarker of pluripotent stem cells,^45^, ^46^ such as embryonic stem cells^47^ and induced pluripotent stem cells.^45^, ^46^, ^48^ Therefore, AP staining is usually used to detect the reprogramming efficiency, the subset of undifferentiated pluripotent stem cells with extensive and likely unrestricted self‐renewal potential in detailed.^49^, ^50^ With the differentiation of cells, AP activity rapidly decreased.^51^ SKP stemness can also be detected with this approach, since they are multipotent stem cells as well.^52^, ^53^

### 2.6 Skin embedding and haematoxylin and eosin (H&E) staining

The skin was collected, fixed with 4% PFA for 24 hours and transferred to 70% ethanol. After fixation, samples were embedded in paraffin and sectioned (10‐μm thickness), with the sections stained using an H&E staining kit (Abcam) according to standard procedures.

### 2.7 HF reconstitution

Female BALB/c ^nu/nu^ mice (4‐week‐old) were anaesthetized with sodium pentobarbital (40 mg/kg) via intraperitoneal injection, and skin wounds (symmetrical, full‐thickness, 5‐mm diameter) were created on the disinfected back using a sterile and disposable 5‐mm biopsy punch (cat no. 50.005; Gyneas). Treated or untreated SKPs (2 × 10^6^) and newborn‐mouse‐derived Epi‐SCs (1 × 10^6^) were encapsulated in 30 μL of thawed Matrigel (Corning Inc), and the cell‐matrigel mixture was incubated at 37°C for 30 minutes prior to implantation into the full‐thickness excisional wound. The wound was then covered with a tegaderm transparent dressing (2‐3/8 × 2‐3/4 inches; 3 M, St. Paul, MN, USA) and a self‐adhering elastic bandage (2.20 yards; Johnson & Johnson). After 3 weeks, the newborn hair was photographed under a stereoscopic microscope (SZM7045TR; Keyence), and the mice from whom hair samples were obtained for photographing, fixing and paraffin‐embedding were sacrificed.

### 2.8 Immunofluorescence (IF) staining

The skin or SKPs were fixed and processed for IF staining, as described previously,^54^ to determine levels of AREG, the proliferation marker protein Ki‐67, keratin‐14 (K14) and phosphorylated EGF receptor (p‐EGFR). The samples were photographed under an inverted laser scanning confocal microscope (LSM880 with Airyscan; Carl Zeiss).

### 2.9 Western blot analysis

Briefly, cells were lysed with radioimmunoprecipitation assay lysis buffer (Beyotime), and cell lysates were subjected to 8% sodium dodecyl sulphate polyacrylamide gel electrophorese. Primary antibodies for p‐EGFR, EGFR, p‐phosphoinositide 3‐kinase (PI3K), PI3K, p‐RAC‐α serine/threonine‐protein kinase (AKT), AKT, p‐mitogen‐activated protein kinase kinase (MEK), MEK, p‐mitogen‐activated protein kinase (MAPK), MAPK, p‐(ETS domain‐containing protein) ELK, ELK, glyceraldehyde 3‐phosphate dehydrogenase (GAPDH) and AREG (Table S3) were detected using horseradish peroxidase‐conjugated anti‐rabbit or anti‐mouse secondary antibodies (Table S4) with an enhanced chemiluminescence reagent (Advansta).

### 2.10 Transcriptome RNA sequencing (RNA‐seq) analysis

Purified total RNA from SKPs was used for RNA‐seq library preparation. For mRNA library construction, purified mRNA was fragmented into small pieces with fragment buffer at an appropriate temperature, followed by generation of first‐strand cDNA using random hexamer‐primed reverse transcription and second‐strand cDNA synthesis. To end the repair, A‐tailing mix and RNA index adapters were added, and the acquired cDNA fragments were amplified by polymerase chain reaction (PCR), purified using Ampure XP beads (Beckman Coulter), dissolved in elution buffer and validated using a 2100 bioanalyzer (Agilent Technologies) for quality control. The double‐stranded PCR products were heated and circularized using the splint oligo sequence to obtain the final library. Single‐strand circular DNA was formatted as the final library and amplified with phi29 DNA polymerase to create a DNA nanoball, with ≥300 copies of one molecule. The qualified products were then loaded into the patterned nanoarrays and processed for 50‐bp paired‐end sequencing on a BGISEQ‐500 sequencer (BGI‐Shenzhen). After filtering with SOAPnuke (v1.5.2),^55^ clean reads were obtained and stored in FASTQ format and then aligned to the mouse genome using HISAT2 (v2.0.4).^56^ Bowtie2 (v2.2.5)^57^ was used to map the clean reads to the reference coding gene set, and gene‐expression levels from different samples were calculated using RSEM (v1.2.12).^58^ Differential expression analysis was performed using DEGseq2 (v1.4.5)^59^ and a *Q*‐value ≤0.05. To observe phenotypic changes, gene ontology (GO) and kyoto encyclopaedia of genes and genomes (KEGG) enrichment analyses of annotated differentially expressed genes (DEGs) were performed by phyper (https://stat.ethz.ch/R‐manual/R‐devel/library/stats/html/Hypergeometric.html) to determine hypergeometric distributions. Significance levels of terms and pathways were corrected according to a rigorous threshold (*Q*‐value ≤0.05) using the Bonferroni test.^60^

### 2.11 Telogen‐to‐anagen transition assay

Mice were randomly divided into eight groups: control, AREG (0.5 mg/kg body weight), Wortmannin (0.5 mg/kg body weight), AREG + Wortmannin, PD98059 (3 mg/kg body weight), AREG + PD98059, Wortmannin + PD98059 and AREG + Wortmannin + PD98059. AREG or inhibitors were multipointedly subcutaneously injected into the dorsal skin of 56‐day‐old mice as grouped, whereas the control mice received PBS. At 10‐days post‐injection, the dorsal skin was photographed under a stereoscopic microscope (SZM7045TR) to assess changes in telogen‐to‐anagen transition, and the regenerated HFs were evaluated by IF for Ki‐67^+^ cells.

### 2.12 Statistical analysis

All statistical analyses were performed using GraphPad Prism software (v.7.0; GraphPad Software) and Origin Pro‐2018 software (OriginLab). Statistical significance between two groups was measured using an unpaired *t* test. One‐way analysis of variance (ANOVA) was used to compare three or more groups. All data are expressed as the mean ±standard error of the mean (SEM; n ≥ 3), with a *P* < .05 considered significant.

## 3 RESULTS

### 3.1 Epi‐SCs and ASCs promote SKP stemness in vitro and in vivo

**FIGURE 1:**
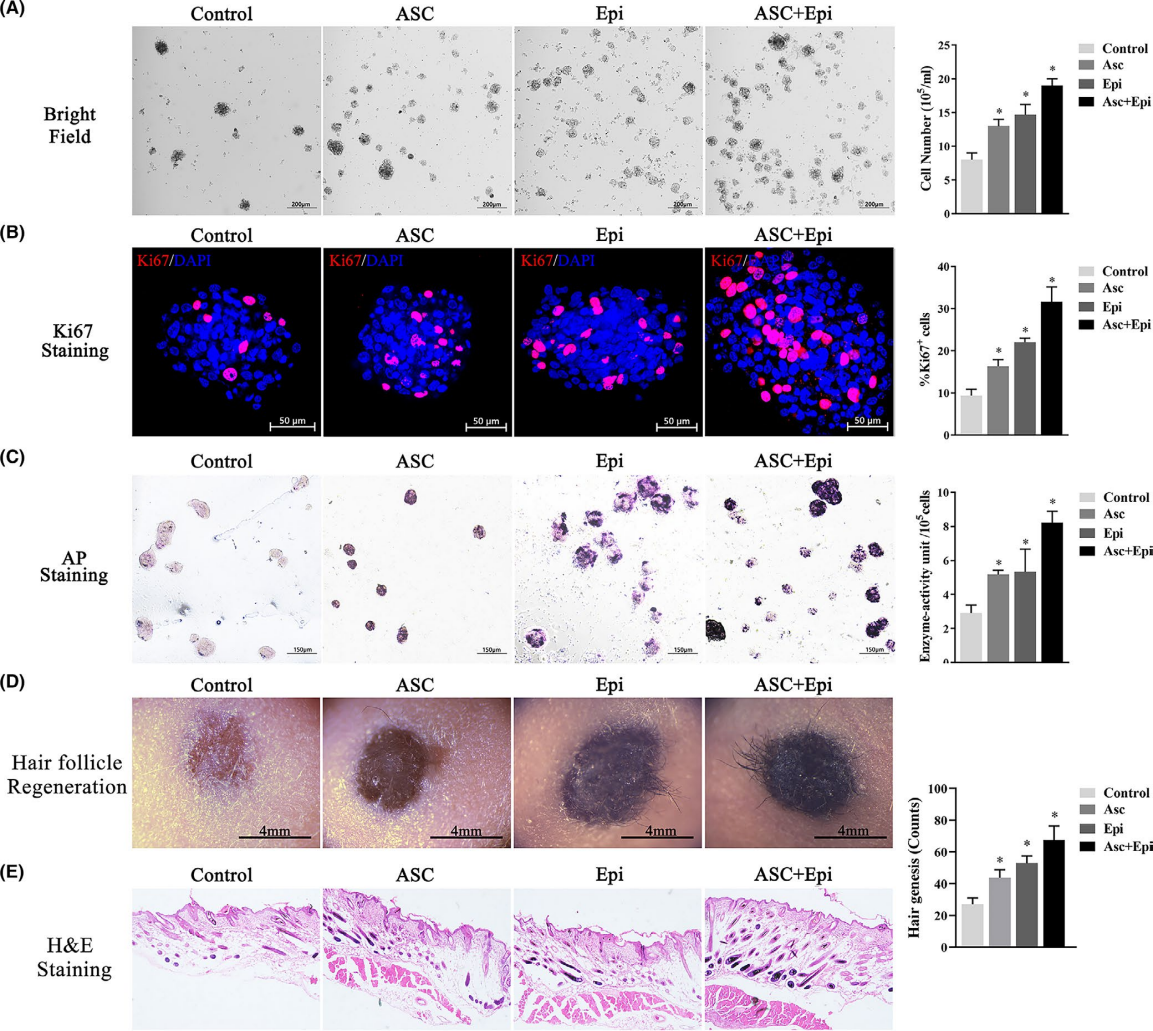
Co‐culture of SKPs (P3) for 6 d. (A) Bright‐field image showing cell expansion when cultured alone or in the presence of ASCs, Epi‐SCs or both. (B) The percentage of Ki‐67^+^cells, with regions of active proliferation highlighted. (C) AP staining of SKPs. (D) Formation of HFs in composites with treated SKPs and Epi‐SCs grafted onto BALB/c^‐nu/nu^ after 21 d. (E) H&E staining of skin cross‐sections from four groups of BALB/c^‐nu/nu^ mice. *P < .05 vs control

To determine whether the co‐culture system promotes SKP stemness, we divided the SKPs (P3) into four groups: the SKP group, the SKP and ASC group (the SA group); the SKP and epidermal stem cells (Epi‐SCs) group (the SE group); and the group with SKPs, ASCs and Epi‐SCs (the SAE group). We then determined cell numbers, levels of the proliferation marker Ki‐67 and AP activity. As expected, we observed increased cell numbers (Figure 1A), proliferation ratio (Figure 1B) and AP activity (Figure 1C) in the SAE group relative to the SA and SE groups, with SKPs cultured alone showing the lowest indices. In the meantime, we exploited the AP Assay Kit for the absolute quantification of their enzyme activity, with results depicted on the right‐hand‐side picture (Figure 1C). Additionally, the results of the HF‐reconstitution experiment in vivo confirmed that co‐cultured SKPs increased the number of regenerated HFs in vivo (Figure 1D), and H&E staining (Figure 1E) showed intact structures without abnormalities and consistent hair‐morphogenic trends with previous results. These results indicated that co‐culturing with Epi‐SCs and ASCs promoted SKP stemness both in vitro and in vivo.

### 3.2 The co‐culture system activates the ErbB pathway of SKPs

**FIGURE 2:**
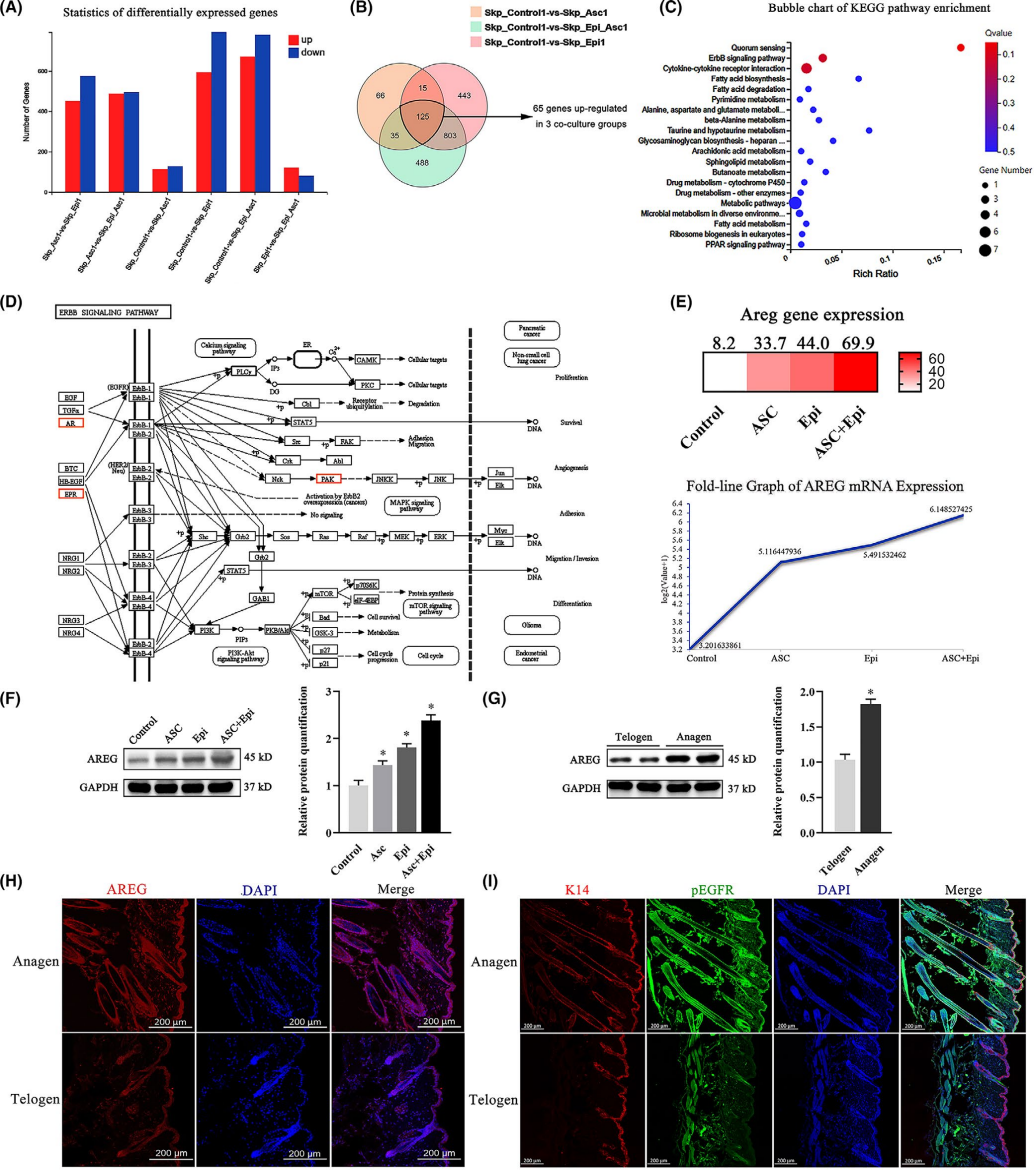
Statistical analysis of changes in SKP gene expression. (A) DEGs in SKPs (P3) after 6‐d co‐culture or culture alone. (B) Venn diagram showing the overlap of DEGs from three co‐culture groups vs control. (C) Bubble chart showing the top 20 enriched pathways according to KEGG pathway analysis for 65 upregulated genes. (D) KEGG pathway map for the ErbB signalling pathway. The upregulated targets of the ErbB pathway are highlighted in orange. (E) Heat map and fold‐line graph of *AREG* expression. (F) Analysis of AREG levels by Western blot at 6 d after co‐culture or culture alone. (G) AREG expression in HFs during the telogen stage (19‐d‐old mice) and anagen stage (35‐d‐old mice) according to Western blot. (H, I) AREG, K14, p‐EGFR and DAPI IF of HFs in the same anagen stage and telogen stage. *P < .05 vs control

To understand the contribution of each cell type to the SKPs in the co‐culture system, we performed transcriptome profiling by RNA‐seq on SKPs from different groups. Figure 2A shows statistical comparisons of DEGs between the different groups, with venn diagrams (Figure 2B) revealing overlapping DEGs from the three co‐culture groups vs the control and identifying 241 genes from the SA group, 1386 genes from the SE group and 1451 genes from the SAE group showing significantly alterations in SKPs. Following KEGG pathway analysis and GO‐Term analysis for the 65 upregulated genes among the significant DEG sets (125 genes) between the three groups, the top 20 enriched canonical pathways are depicted in Figure 2C. KEGG enrichment analysis using a false discovery rate *Q*‐value ≤0.05 revealed the ErbB pathway among all of those upregulated (Figure 2D) as having a low Q‐value and close relationship with cell proliferation, survival, adhesion and differentiation, thereby suggesting its potential as a critical pathway in promoting SKP stemness in the co‐culture system. Moreover, RNA‐seq results indicated three significantly upregulated genes in this pathway: *AREG*, *epiregulin* and *p21‐activated kinase 3*. Given its consistent expression trend (Figure 2E) correlating with SKP proliferation, we chose *AREG* for subsequent experimental analysis.

We then investigated AREG‐expression patterns using Western blot and IF. Co‐culturing with Epi‐SCs or ASCs increased AREG levels in SKPs (P3) relative to levels in control cells, with SKPs in the SAE group showing the highest AREG expression (Figure 2F). To further demonstrate the role of AREG in HF development, we assessed AREG‐expression patterns, revealing that levels were higher during the anagen stage than during the telogen stage (Figure 2G,H, Figure S1). Among the ErbB family of receptors, AREG has a high binding affinity for EGFR. To determine whether receptor activation by AREG occurred in the anagen phase, we investigated levels of p‐EGFR, with the result exhibiting consistent changes in levels along with those of AREG, as expected (Figure 2I, Figure S2). These results implied that the co‐culture system activated the ErbB pathway and upregulated AREG expression in SKPs, leading to hair morphogenesis in the anagen phase by activating EGFR.

### 3.3 AREG promotes SKP stemness in vitro and in vivo

**FIGURE 3:**
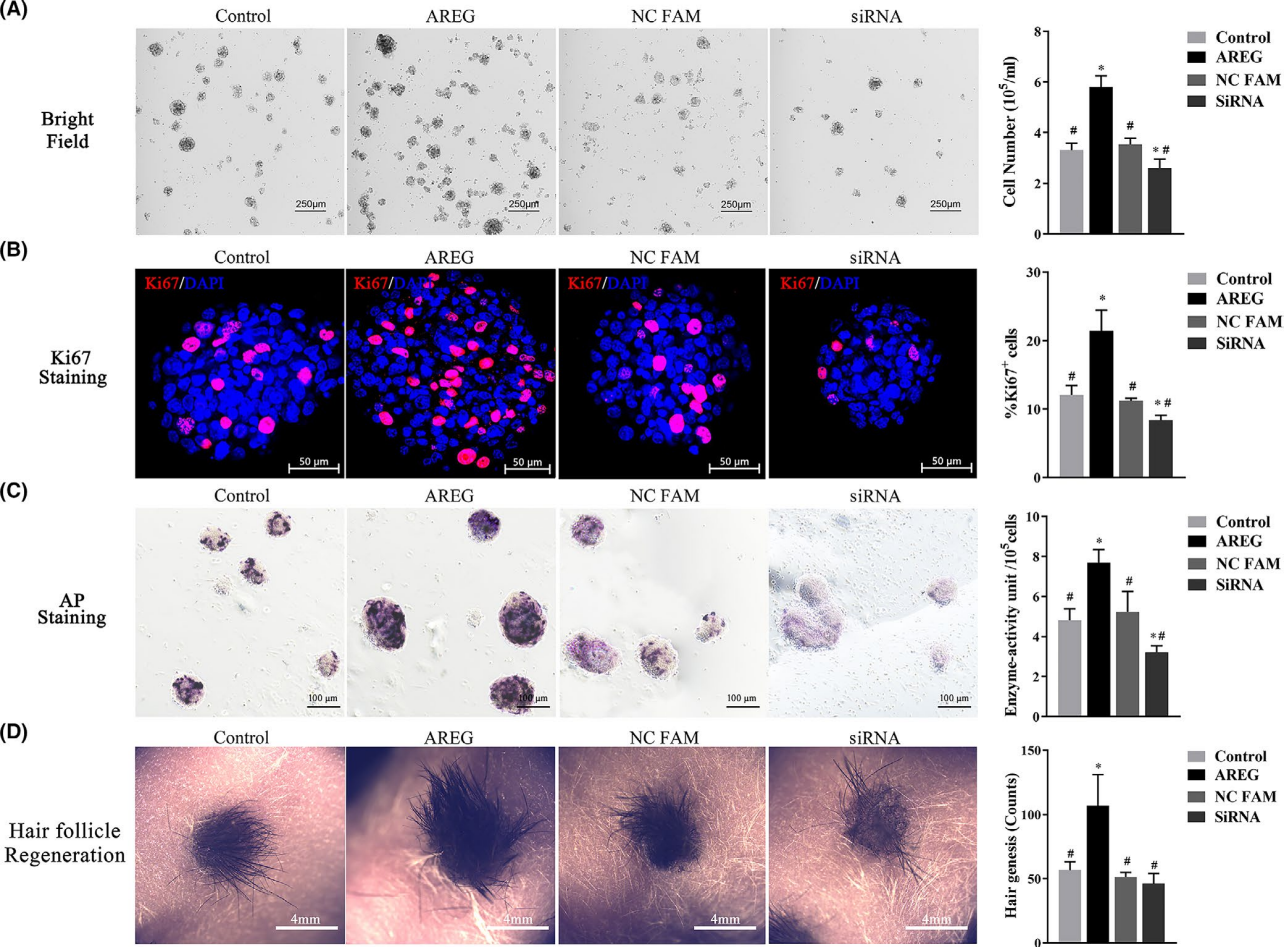
Analysis of SKP stemness according to AREG expression. SKPs (P0) were divided into groups and treated with AREG (80 ng/mL) for 3 d, co‐transfected with either control siRNA (200 nmol/L) or *AREG* siRNA (200 nmol/L) for 48 h, or normally cultured as the control group. The medium was replaced with fresh medium, and cells were grown for an additional 24 h before analysis. The effects of AREG supplementation or AREG knockdown on SKP stemness are indicated by (A) cell number, (B) Ki‐67 staining and (C) AP staining. (D) HF regeneration in composites of treated SKPs and Epi‐SCs grafted onto BALB/c‐nu/nu after 21 d. #*P* < .05 vs AREG treatment; *P < .05 vs control

To validate the role of AREG in enhancing SKP stemness, we divided SKPs (P3) into four groups: control, AREG, negative‐control FAM (NC‐FAM) and siRNA. We then conducted AP and Ki‐67 staining for in vitro experiments, as well as HF reconstitution for in vivo experiments. The SKPs in the AREG group were cultured in DMEM‐EFB with 80 ng/mL AREG, those in the NC‐FAM group were cultured in DMEM‐EFB supplemented with control siRNA, and the siRNA group involved *AREG* silencing for 48 hours. As shown in Figure 3, AREG significantly increased the cell number (Figure 3A) and percentage of Ki‐67^+^ SKPs (Figure 3B) (both signs of proliferative capacity), as well as AP activity (Figure 3C) (demonstrating SKP pluripotency), whereas AREG knockdown had the opposite effect. Furthermore, the HF‐reconstitution assay (Figure 3D) showed that the AREG group demonstrated the best hair neogenesis, and that the siRNA group displayed the worst among the four groups, thereby indicating that AREG promoted SKP stemness both in vitro and in vivo.

### 3.4 AREG activates the PI3K and MAPK pathways in vitro

**FIGURE 4:**
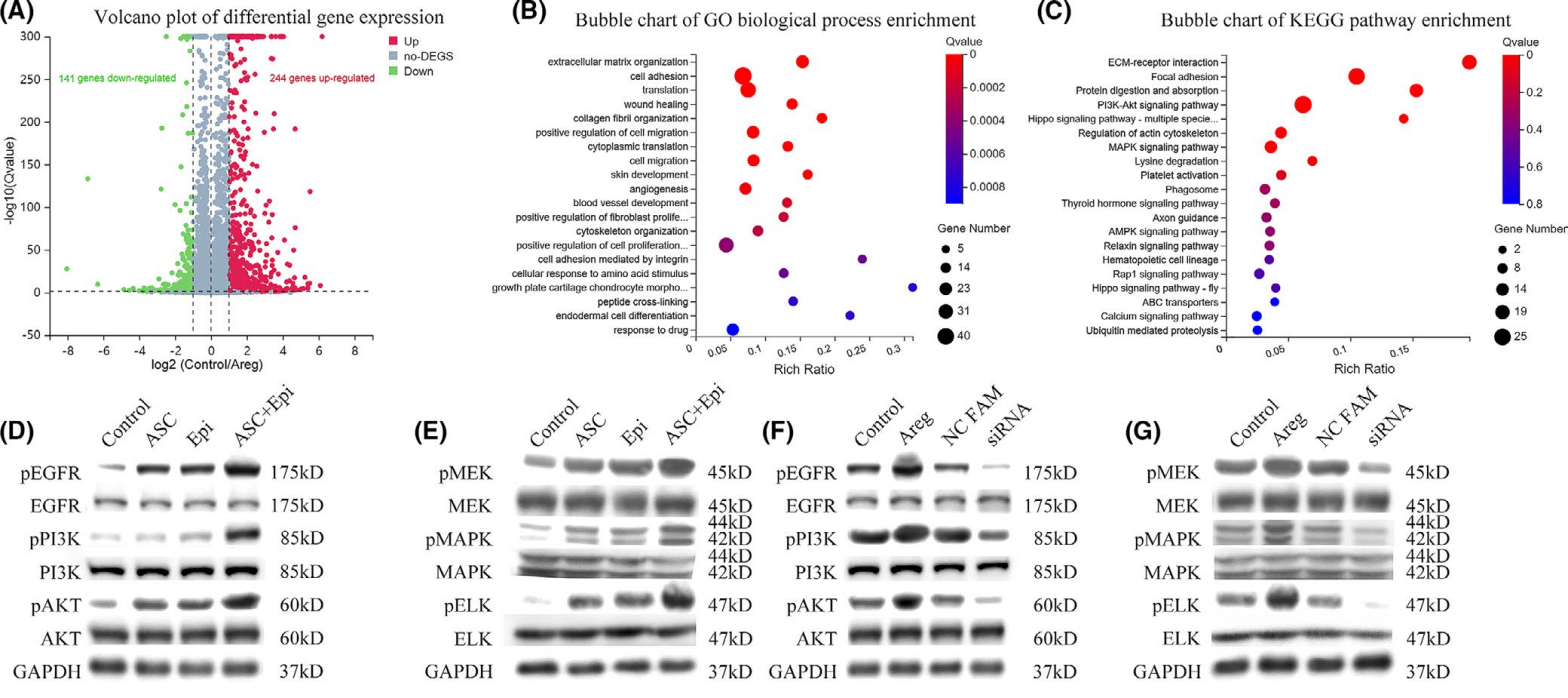
Measurement of differential gene expression following AREG supplementation. Analysis of DEGs between the control group and the group receiving treatment with 80 ng/mL AREG for 3 d. (A) Volcano plots showing upregulated and downregulated genes. (B) Bubble plot showing differential enrichment of 244 upregulated genes according to GO biological process categories (top 20 are presented). (C) Bubble chart showing the top 20 enriched KEGG pathways for the 244 upregulated genes. (D‐G) Protein levels in the PI3K and MAPK pathways according to Western blot

To determine which signalling pathway downstream of EGFR is activated, we performed global transcriptional profiling, followed by next‐generation RNA‐seq analysis of SKPs treated with AREG (80 ng/mL for 3 days) or cultured alone. As shown in Figure 4A, AREG‐treated SKPs exhibited 244 upregulated genes and 141 downregulated genes. GO and KEGG analyses of the 244 upregulated genes identified skin development and positive regulation of cell‐population proliferation as enriched processes (Figure 4B). Besides, the MAPK and PI3K signal‐transduction pathways, which are highly involved in cell growth, proliferation, survival and differentiation,^61^ were enriched according to KEGG analysis (Figure 4C). The separately enriched genes from the two pathways are listed in Table S2. SKPs undergoing AREG treatment or knockdown, co‐culturing with both Epi‐SCs and ASCs or either one of them, and culturing alone were then lysed to determine changes in phosphorylation levels of EGFR, PI3K, AKT, MEK, MAPK and ELK between the two pathways, revealing that phosphorylation levels matched the observed enhancement of SKP stemness from AREG or co‐culture treatment (Figure 4D‐G). These results indicated that AREG played an essential role in the co‐culture system through the PI3K and MAPK pathways in vitro.

### 3.5 AREG promotes SKP stemness in vitro through the PI3K and MAPK pathways

**FIGURE 5:**
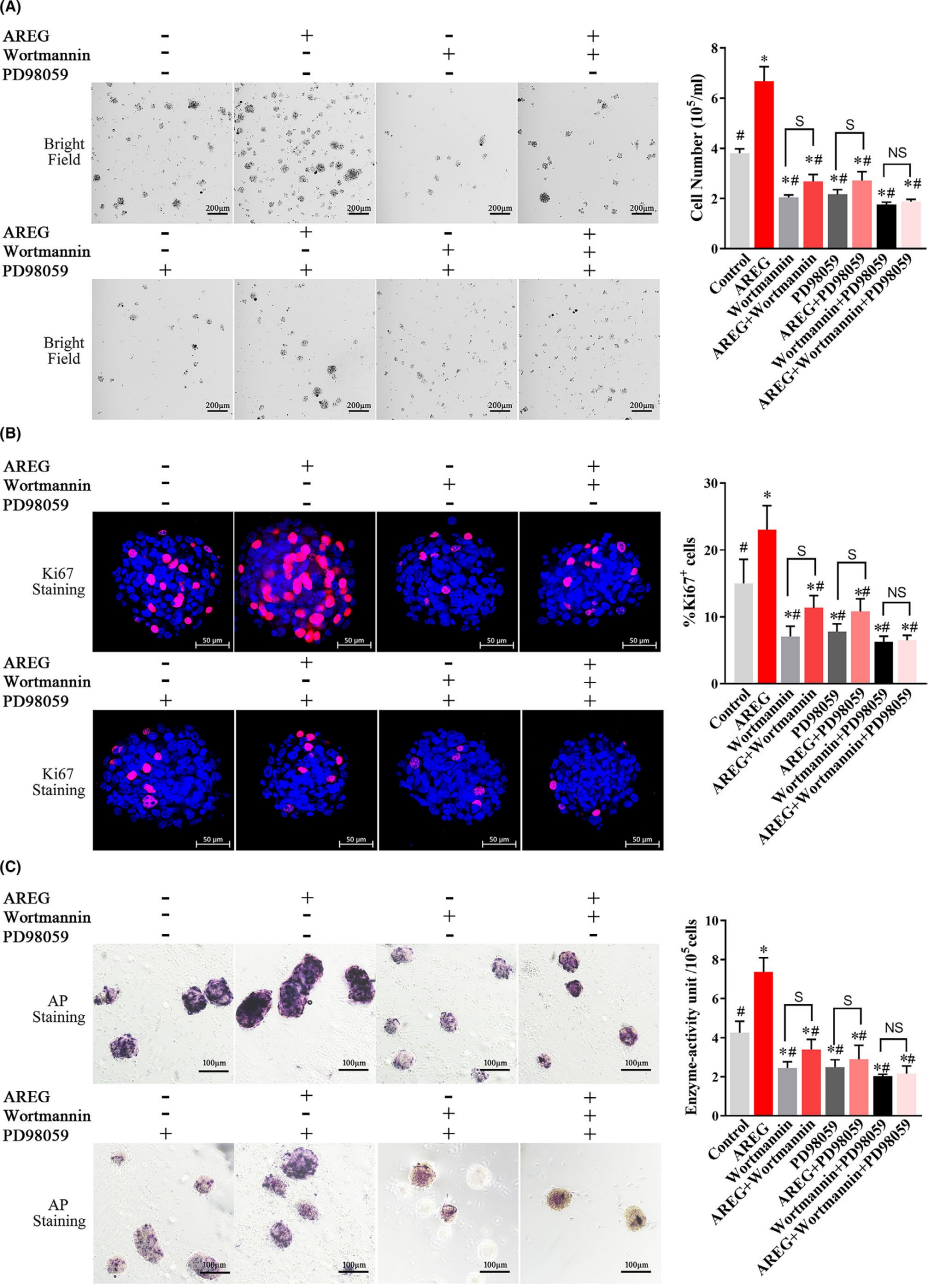
Amphiregulin promotes SKP stemness in vitro through the PI3K and MAPK pathways. Evaluation of PI3K (wortmannin, 100 nmol/L) and MAPK (PD98059, 50 μmol/L) inhibition alone or together in the presence of AREG (80 ng/mL) or not in vitro for 3 d. Changes in SKP stemness were evaluated according to (A) changes in cell number, (B) Ki‐67 staining, and (C) AP staining. #*P* < .05 vs AREG treatment; *P < .05 vs control. S, significant difference; NS, not significant

To verify the role of AREG‐mediated PI3K and MAPK signalling in SKP stemness, we classified SKPs (P3) into eight groups: control, AREG, wortmannin, AREG + wortmannin, PD98059, AREG + PD98059, wortmannin + PD98059 and AREG + wortmannin + PD98059, which were cultured for 3 days. Administration of PI3K inhibitor (wortmannin, 100 nmol/L) or MAPK inhibitor (PD98059, 50 μmol/L) significantly decreased SKP cell number (Figure 5A), ratios of Ki‐67^+^ cells (Figure 5B) and AP activity (Figure 5C). Notably, AREG treatment slightly rescued the adverse effects of wortmannin or PD98059 alone; however, addition of both inhibitors completely blocked AREG‐mediated promotion of stemness.

### 3.6 AREG induces the telogen‐to‐anagen transition in vivo through the PI3K and MAPK pathways

**FIGURE 6:**
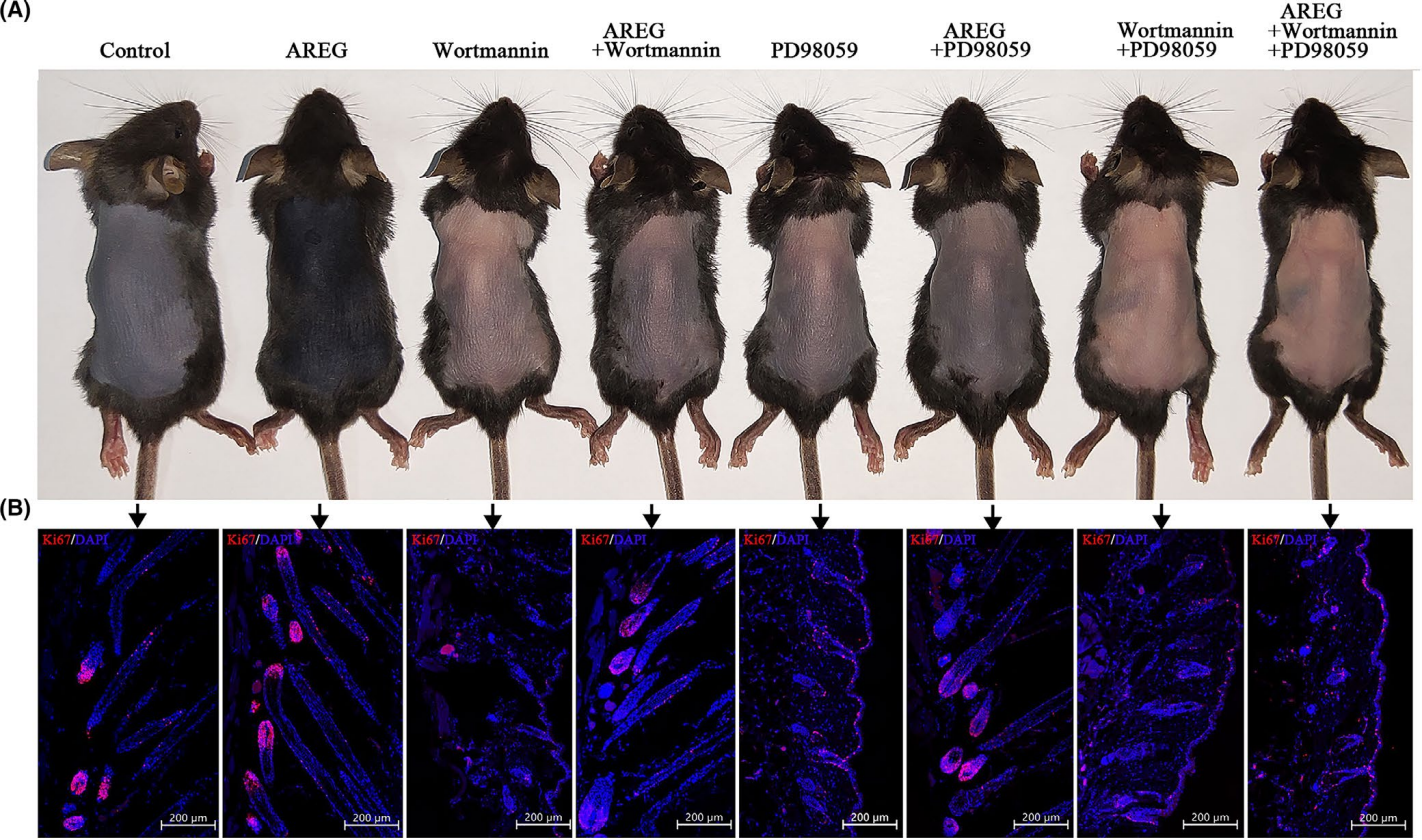
Amphiregulin induces the telogen‐to‐anagen transition in vivo through the PI3K and MAPK pathways. Mice were randomly divided into eight groups, and AREG (0.5 mg/kg body weight, every 48 h), Wortmannin (0.5 mg/kg body weight, every 48 h), and PD98059 (3 mg/kg body weight, every 48 h) were subcutaneously injected into the dorsal skin of 56‐d‐old C57 mice, whereas controls received PBS. At 10‐d post‐injection, (A) the dorsal skin was photographed under a stereoscopic microscope to assess changes in telogen‐to‐anagen transition, and (B) HFs were evaluated by IF for Ki‐67^+^ cells

To evaluate the hair‐promoting effect of AREG in vivo, multipoint subcutaneous injection of AREG (0.5 mg/kg body weight, every 48 hours) with or without one or both of the downstream pathway inhibitors [PI3K (wortmannin, 0.5 mg/kg body weight, every 48 hours) and MAPK (PD98059 [3 mg/kg body weight, every 48 hours]) to the shaved dorsal skin of 56‐day‐old C57BL/6 mice was performed, and skin pigmentation and hair growth were observed (Figure 6A), Ki‐67^+^ cells evaluated in HFs (Figure 6B, Figure S3). Compared with NC mice, AREG induced an earlier transition from telogen stage to anagen one along with a higher number of Ki‐67^+^ cells in the DP. However, after applying one or both of the inhibitors, HF entry to the anagen stage was delayed, which could not be reversed by AREG. All in vivo outcomes were consistent with the in vitro results (Figure 5). These results suggested that AREG induced stemness in vitro and the telogen‐to‐anagen transition in vivo through the PI3K and MAPK pathways.

## 4 DISCUSSION

This study used 3D co‐culture models to mimic the in vivo microenvironment and concluded that AREG plays an essential role in enhancing the stemness of SKPs through the PI3K and MAPK pathways in vivo and in vitro. These findings suggest AREG as a promising therapeutic strategy for alopecia.

Based on the application of the co‐culture system to remedy the shortcomings of monoculture fermentation and increase the biosynthetic efficiency of natural products,^62^ we successfully generated highly proliferative spherical SKPs by establishing a ternary‐cell 3D co‐culture system. In this system, Epi‐SCs, SKPs and ASCs are separately plated in three‐layer chambers mimicking skin epidermis, dermis and hypodermis in order to facilitate the diffusion of small molecules between inserts and divide cells into their designated tiers without physically contacting with one another. The 2D model is thus not able to simulate such a normal anatomical position. Upon addition, the 2D culture settings hinder us from isolating the SKPs from other cells, posing the subsequent difficulties for the separate analysis for SKPs.

Moreover, the SKPs are isolated via an adherence‐separation method, which keeps them separate from Epi‐SCs and fibroblasts.^63^ In the first 8 hours after separation, Epi‐SCs readily adhered to the bottom of the well^52^ and did not generate spherical colonies under SKPs first culture conditions described in the methods section. Secondary clonality of the spheres expressing SKPs markers was subsequently formed from dissociated primary spheres, acquisition of which was confirmed according to a growing body of evidence from other 3D colony‐forming systems (eg, methylcellulose or matrigel).

Following this period, premature adherence has typically resulted in a reduced SKPs yield, owing to the fact that the SKPs in the adherent sphere will immediately differentiate into dermal fibroblasts and gradually lose their stemness.^52^ Therefore, some studies utilized rolling cultures or gentle agitation to promote sphere formation.^52^ Besides, Ohsang Kwon et al observed that the 3D skin equivalent culture assay reproducibly regenerated hair follicles while SKPs precultured in the 2D model with epidermal cells did not. Also, several representative genes related to hair induction, such as AP, Hey1, versican (hair‐inducing properties),^64^ CD133,^65^ TBX18^66^ (markers of DP precursors) and Sox2 (the highest‐expressed transcription factors in the DP)^67^, ^68^ showed higher expression in 3D culture assay than in the 2D chamber assay.

Utilizing this co‐culture model and subsequent RNA‐seq analysis, we discovered that the expression of AREG in SKPs was driven by Epi‐SCs and ASCs. Previous studies report that AREG, a widely expressed transmembrane tyrosine kinase of the EGF family,^69^ behaves as autocrine,^70^ paracrine^71^ and juxtacrine^71^, ^72^ factors involved in a broad range of physiological processes that trigger central intracellular signalling cascades governing proliferation, survival, motility, angiogenesis and inhibition of apoptosis.^73^, ^74^ Soluble AREG presents a C‐terminal domain that shares significant overall homology with all other EGF‐like growth factor family members. After binding to a ligand, EGFR homo‐ or heterodimerizes with ErbB2, ErbB3 or ErbB4,^75^ leading to autotransphosphorylation of the tyrosine kinase domain and activation of a complex network of pathways, including those associated with MAPK, PI3K, phospholipase Cγ, and signal transducer and activator of transcription pathways.^76^ One remarkable determinant of specific triggers of corresponding signals in the EGFR system is its differential spatiotemporal expression in response to a given stimulus, which is observed in various physiological phenomena related to AREG. For example, AREG is the predominant EGFR ligand upregulated in skin mimicking cutaneous injury models, where heparin‐binding EGF upregulation was followed by that of AREG in promoting wound healing through a potent stimulus of keratinocyte proliferation in skin homeostasis.^77^, ^78^, ^79^ Additionally, AREG was identified as the ascendant EGFR ligand upregulated during blastocyst implantation,^80^, ^81^ and mammary gland, neuronal, bone and trophoblast development.^82^, ^83^, ^84^ Furthermore, AREG does not result in EGFR degradation in contrast to EGF, an effective trigger of EGFR degradation,^85^ but rather targets EGFR towards a recycling pathway^86^ and promotes EGFR accumulation.^87^ These findings suggest AREG as a promising target for promoting EGFR signalling in skin restoration; however, no study had previously demonstrated the influence of AREG on dermis‐derived cells, such as SKPs.

Therefore, the present study determined whether AREG improves SKP stemness and the associated mechanisms. We employed RNA‐seq rather than cDNA microarrays, because RNA‐seq allows assessment of the presence and quantity of RNA transcripts from whole‐genome samples, whereas cDNA microarrays only identify finite allele variants designed into the microarrays. Additionally, RNA‐seq shows higher resolution for detecting changes in gene expression relative to cDNA microarrays.^88^ Following acquisition of RNA‐seq results, GO^89^ and KEGG^90^ enrichment analyses were performed to identify enriched metabolic pathways and/or biological processes, which subsequently identified involvement of the PI3K and MAPK pathways.

Both the PI3K and MAPK pathways played a critical role in multiple cellular responses elicited by AREG signalling, which agreed with our previous findings reporting involvement of the PI3K pathway in wound‐induced HF telogen‐to‐anagen transition^91^ and a vital pathway for epidermal and dermal cell communication, which is indispensable for HF regeneration.^54^ Moreover, the PI3K pathway is involved in tissue regeneration, as highlighted by the reported decline in the long‐term regeneration capability of hematopoietic stem cells in Akt knockout mice,^92^ whereas *phosphatase and tensin homolog* knockout in Lgr5^+^ HF stem cells enhanced HF regeneration after wounding.^54^ Importantly, the PI3K pathway inhibits senescence and promotes the self‐renewal of human SKPs *in vitro*.^93^ Regarding the MAPK pathway, EGFR stimulation provokes MAPK in a multistep process, which results in transcription factor translocation to the nucleus and alters gene expression to promote growth, proliferation and/or differentiation^94^ and described by the effect of lysophosphatidic acid on fibroblasts.^95^ Moreover, previous studies suggest that the MAPK pathway cooperates with PI3K and Rac1 signalling to induce DNA synthesis.^96^, ^97^ These findings, as well as those of the present study, suggest a critical role for AREG in HF regeneration and its potential efficacy as a therapeutic approach for de novo HF regeneration.

This study has limitations. First, we were unable to elucidate the precise mechanism associated with the AREG‐mediated activity; therefore, further studies are necessary to determine the transcription factors responsible for the SKP‐specific expression profile observed following co‐culture with the Epi‐SCs and ASCs. Second, there is overwhelming evidence supporting a role for AREG in tumour development, as AREG is upregulated in numerous neoplasms, including head, neck, lung, breast, stomach, liver, colon, prostate, bladder and skin tumours,^73^ and capable of self‐sufficient growth and survival signals.^72^ Moreover, functional studies indicate that AREG can perform as a pro‐oncogenic factor, affecting most cancer hallmarks.^98^ Therefore, a gap exists between experimental and clinical findings regarding AREG‐related roles, and further investigations are required to clarify its efficacy for future clinical applications. Furthermore, caution is needed and safety should be confirmed when extrapolating experimental results to a clinical setting, including the long‐term treatment effects and further in vitro studies in human cells and in vivo experiments in larger animals, which are required to confirm the approaches to inducing SKP stemness in HFs as promising yet underexplored lines of research.

## 5 CONCLUSIONS

We applied a 3D co‐culture model and determined that AREG promoted SKP stemness by enhancing both cell proliferation and hair‐inducing capacity through the PI3K and MAPK pathways, thereby, providing insight into a promising strategy of AREG for de novo hair regeneration in treating alopecia.

## CONFLICT OF INTEREST

The authors declare that they have no conflicts of interest regarding this study.

## AUTHOR CONTRIBUTIONS

Qiumei Lu and Weiwei Chu contributed to the study concept and design. Qiumei Lu, Ying Gao and Zhimeng Fan contributed to acquisition of data. Qiumei Lu, Ying Gao, Zhimeng Fan, Xing Xiao, Yu Chen, Yuan Si, Deqiang Kong, Shuai Wang, Meijian Liao, Xiaodong Chen, Xusheng Wang and Weiwei Chu contributed to analysis and interpretation of data. Qiumei Lu, Ying Gao, Zhimeng Fan, Xing Xiao, Yu Chen, Yuan Si, Deqiang Kong, Shuai Wang, Meijian Liao, Xiaodong Chen, Xusheng Wang and Weiwei Chu contributed to writing, reviewing and approval of the final version of this work.

## Supporting information

App S1

## Data Availability

All data included in this study are available upon request to the corresponding author.
