# Supplementary material for: Amphiregulin promotes hair regeneration of skin‐derived precursors via the PI3K and MAPK pathways
Source: Cell Prolif. 2021 Aug 12;54(9):e13106. doi: 10.1111/cpr.13106 (PMC8450126; doi:10.1111/cpr.13106)

**Appendix S1**

**Supplementary Data**

**Supplementary Table 1. Sequences used to regulate AREG**

| RNA oligo name | Sequences (5′-3′) | |
| --- | --- | --- |
| Areg-Mus-277 | sense | GCCAUUAUGCAGCUGCUUUTT |
|  | antisense | AAAGCAGCUGCAUAAUGGCTT |
| Areg-Mus-351 | sense | GCCGGUGGACUUGAGCUUUTT |
|  | antisense | AAAGCUCAAGUCCACCGGCTT |
| Areg-Mus-465 | sense | CCACAAAUAUCCGGCUAUATT |
|  | antisense | UAUAGCCGGAUAUUUGUGGTT |
| Negative control FAM | sense | UUCUCCGAACGUGUCACGUTT |
|  | antisense | ACGUGACACGUUCGGAGAATT |

**Supplementary Table 2.** **Enriched genes from the PI3K and MAPK pathways**

| **KEGG pathway term id** | **KEGG pathway term desc** | | **Term candidate gene num** | **Rich ratio** | **Q value** | **Enriched genes** |
| --- | --- | --- | --- | --- | --- | --- |
| 04151 | PI3K-Akt pathway | 25 | | 0.062 | 8.30E-10 | Gm2436,Itga1,Col4a1,Col6a1,Col6a2,Col6a3,Colla1,Colla2,Ereg,Flt1,Fn1,Nr4al,Igf1r,Igf2,Itga2,Itga5,Itgav,Itgb3,Lama2,Lamb1,Pdgfra,Pdgfrb,Thbs1, Tnc, Lamc3 |
| 04010 | MAPK pathway | 12 | | 0.036 | 0.018 | Ereg,Flt1,Fos,Nr4a1,Igf1r,Igf2,Pdgfra,Pdgfrb,Flna,Dusp1,Hspala,Flnb |

**Supplementary Table 3. Primary antibodies**

| Name | Company | Cat.no. |
| --- | --- | --- |
| phospho-EGFR (pY1068) | CST | 3777 |
| EGFR | CST | 4267 |
| phospho-PI3K (pY458) | CST | 17366 |
| PI3K | CST | 4257 |
| phospho-AKT (pT308) | CST | 13038 |
| AKT | CST | 4691 |
| phospho-MEK (pS217/221) | CST | 3958 |
| MEK | CST | 9126 |
| phospho-MAPK (pT202/Y204) | CST | 4370 |
| MAPK | CST | 4695 |
| phospho-ELK (S383) | Abcam | ab254139 |
| ELK | Abcam | ab227114 |
| GAPDH | Abcam | ab181602 |
| AREG | Thermal | PA5109404 |
| Ki-67 | CST | 9129 |
| Keratin 14 | Biolegend | 906004 |

**Supplementary Table 4. Secondary antibodies**

| Name | Company | Cat.no. |
| --- | --- | --- |
| Donkey anti-Rat Secondary Antibody, Alexa Fluor 488 | Thermal | A21208 |
| Donkey anti-Mouse Secondary Antibody, Alexa Fluor 555 | Thermal | A31570 |
| Donkey anti-Rabbit Secondary Antibody, Alexa Fluor 647 | Thermal | A31573 |
| Goat anti-Mouse Secondary Antibody, Alexa Fluor 647 | Thermal | A32728 |
| Goat anti-Rabbit Secondary Antibody, Alexa Fluor 488 | Thermal | A11008 |

**Supplementary Figure 1. Fluorescence intensity of AREG**


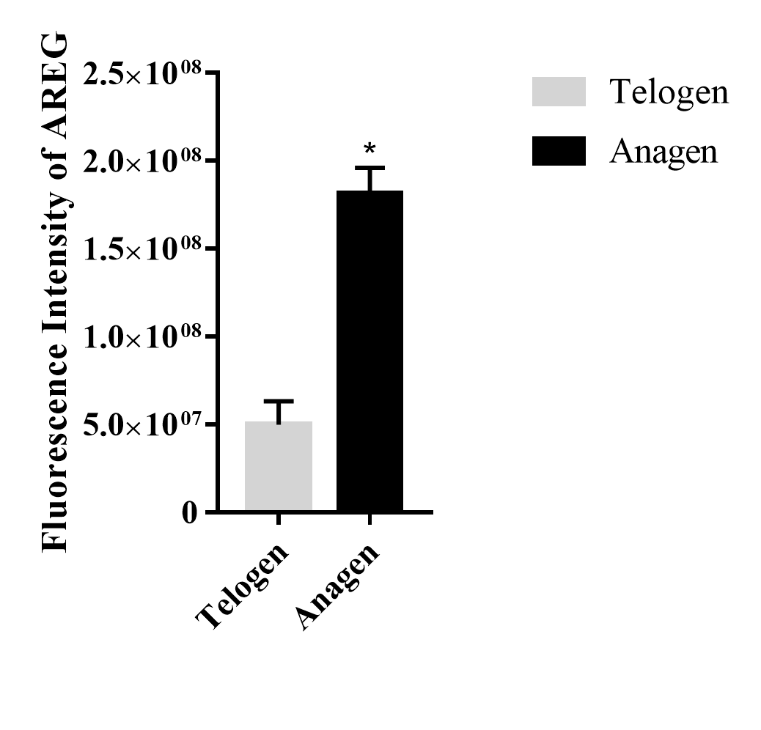


**Supplementary Figure 2. Fluorescence intensity of p-EGFR**


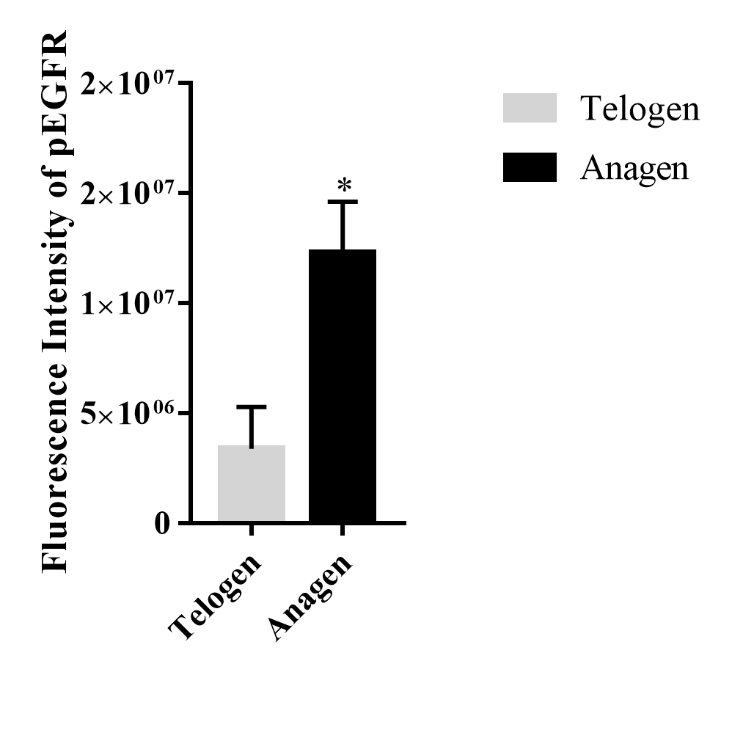


**Supplementary Figure 3. Fluorescence intensity of Ki-67**


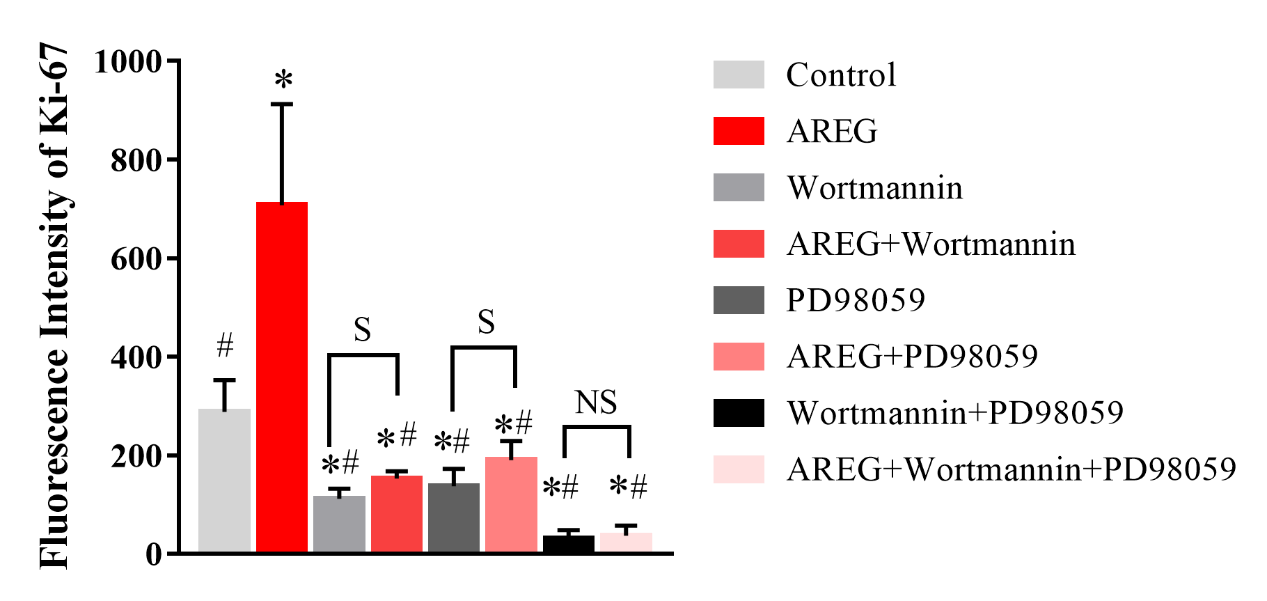

Supplement: Supplementary file 1 — App S1 [file CPR-54-e13106-s001.docx]
